# Genome Sequence of the Oleaginous Yeast Rhodotorula paludigena Strain CM33, a Potential Strain for Biofuel Production

**DOI:** 10.1128/MRA.00286-20

**Published:** 2020-05-07

**Authors:** Chotika Gosalawit, Sumeth Imsoonthornruksa, Natteewan Udomsil, Mariena Ketudat-Cairns

**Affiliations:** a Center for Molecular Structure Function and Application, School of Biotechnology, Institute of Agricultural Technology, Suranaree University of Technology, Nakhon Ratchasima, Thailand; b Division of Food Technology, School of Interdisciplinary Studies, Mahidol University Kanchanaburi Campus, Kanchanaburi, Thailand; Vanderbilt University

## Abstract

The genome sequence of Rhodotorula paludigena strain CM33, an oleaginous yeast isolated from castor bean (*Ricinus* sp.) in Thailand, is reported here. Genome sequencing and assembly yielded 20,657,327 bases with a 64.3% G+C content.

## ANNOUNCEMENT

The nonrenewable nature of fossil fuels has stimulated efforts to find new means of production of non-petroleum-based fuels. Oleaginous yeasts display several advantages over microalgae, fungi, and bacteria. Some genera, such as *Lipomyces* spp., *Rhodosporidium* spp., and *Rhodotorula* spp., are able to accumulate lipid up to 70% of dry cell weight (DCW) (1). Their fatty acid profiles are also similar to those of plants. The red yeast *Rhodotorula* is of interest to the field of applied bioprocessing due to the significant accumulation of fatty acid in the form of triglycerides, which are useful precursors for fatty acid-based biofuels (2, 3). To increase the amount of genomic information and its potential to produce bioenergy from microbial systems, we report here the genome sequence of Rhodotorula paludigena strain CM33.

**Fig. 1:**
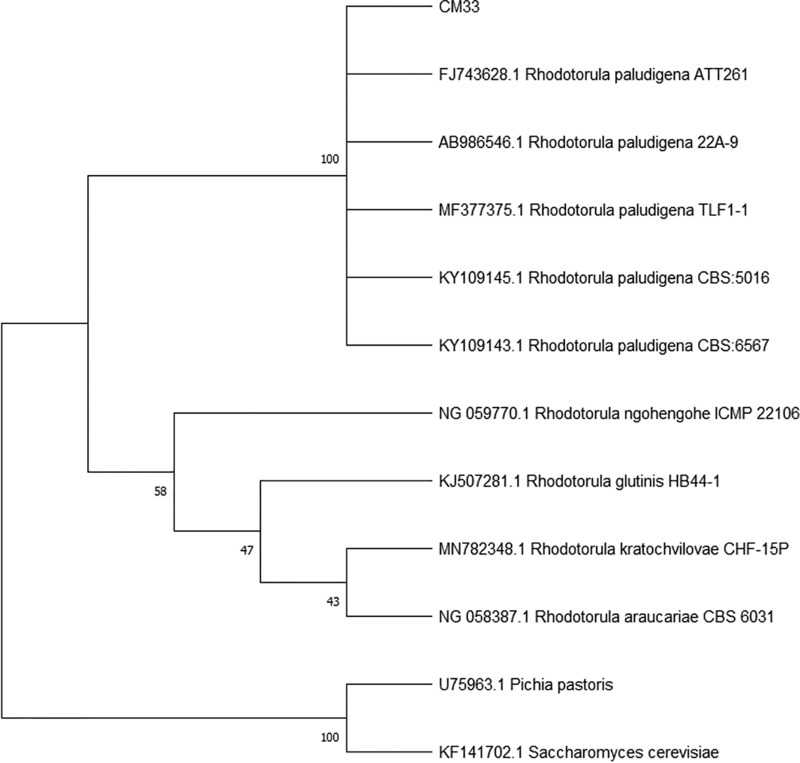
Phylogenetic tree based on D1/D2 26S rDNA sequences displaying phylogenetic relationships between strain CM33 and other members of the genus *Rhodotorula*. The phylogenetic tree was calculated with the maximum likelihood method with 1,000 bootstrap replicates and the Kimura 2-parameter model (8) using MEGA X software (9).

CM33 was isolated from castor beans that were ground and resuspended in 2 ml sterilized deionized (DI) water. Bacterial cell contamination was eliminated by lysis with 200 μl of 10 mg/ml lysozyme, and then dilutions were spread and grown under standard laboratory conditions on yeast extract-peptone-dextrose (YPD) agar (10 g/liter yeast extract, 20 g/liter peptone, 20 g/liter glucose, and 15 g/liter agar) containing 50 μg/ml chloramphenicol (4). Plates were incubated at 30°C for 3 days until colonies were visible. Yeast morphology was observed under a light microscope, and then colonies were restreaked on YPD agar to isolate single colonies. Species identification was performed by sequencing the internal transcribed spacer 5.8S (ITS-5.8S) and domains 1 and 2 (D1 and D2) of the 26S rDNA regions of CM33. To generate these 2 regions, its genomic DNA (gDNA) was extracted with a DNeasy blood and tissue kit (Qiagen, USA) and used as a template. Primers ITS1_F (5′-TCCGTAGGTGAACCTGCGG-3′) and ITS4_R (5′-TCCTCCGCTTATTGATATGC-3′) were used for ITS-5.8S rDNA region amplification, while NL1_F (5′-GCATATCAATAAGCGGAGGAAAAG-3′) and NL4_R (5′-GGTCCGTGTTTCAAGACGG-3′) (5) were used for D1/D2 of 26S rDNA region amplification. The two fragments were cloned into pTG19-T cloning plasmid (Vivantis, Malaysia) and then sequenced by Macrogen, Inc. (South Korea). The ITS-5.8S (GenBank accession number MT279493) and D1/D2 26S (MT279506) sequences from strain CM33 displayed 99.8% identity with those of *Rhodotorula paludigena* (LC190825.1 and KY109146.1, respectively) in a BLAST search against the NCBI database. Phylogenetic analysis revealed that CM33 was grouped in the same cluster as *Rhodotorula paludigena* (Fig. 1).

The gDNA of CM33 was extracted from a 5-ml YPD overnight culture using the Wizard kit (Promega, USA) following the manufacturer’s protocols. The gDNA was quantified using a Qubit assay with a high-sensitivity kit (Life Technologies, USA). The libraries were constructed using the NEBNext DNA library prep master mix kit for Illumina (New England BioLabs, Inc., USA). The DNA was sequenced on an Illumina HiSeq 2000 platform using 2 × 150-bp paired-end reads at Novogene Bioinformatics Technology Co. Ltd. (Hong Kong), leading to the generation of 29,333,333 paired-end reads. Prior to the *de novo* assembly, paired-end reads with low-quality (Q value of ≤38) nucleotides exceeding 40 bp, N-nucleotides exceeding 10 bp, and reads with an adapter overlap exceeding 15 bp were removed from the raw data. After adapter filtering and quality trimming, *de novo* assembly of high-quality clean reads was performed with SOAPdenovo v2.04 (6, 7). For all software, default parameter values were used unless otherwise stated. The genome size was 20,657,327 bp, and it had a G+C content of 64.3%. There were 82 contigs with an *N*_50_ value of 371,695 bp, an *N*_90_ value of 126,264 bp, a maximum contig length of 1,647,824 bp, and a minimum contig length of 20,441 bp.

### Data availability.

The whole-genome shotgun project has been deposited at DDBJ/ENA/GenBank under the BioProject PRJNA491831, BioSample SAMN10089541, and accession number SWEA00000000. The assembled genome sequences are provided under the GenBank accession numbers SWEA01000001 to SWEA01000078. The version described in this paper is the first version, SWEA01000000. The raw data sequences have been deposited in the SRA database under accession number SRX6085390.

## References

[1] Sitepu IR, Garay LA, Sestric R, Levin D, Block DE, German JB, Boundy-Mills KL 2014 Oleaginous yeasts for biodiesel: current and future trends in biology and production. Biotechnol Adv 32:1336–1360. doi:10.1016/j.biotechadv.2014.08.003. 25172033

[2] Kot AM, Błażejak S, Kurcz A, Gientka I, Kieliszek M 2016 *Rhodotorula glutinis*: potential source of lipids, carotenoids, and enzymes for use in industries. Appl Microbiol Biotechnol 100:6103–6117. doi:10.1007/s00253-016-7611-8. 27209039 PMC4916194

[3] Gientka I, Gadaszewska M, Błażejak S, Kieliszek M, Bzducha-Wróbel A, Stasiak-Różańska L, Kot AM 2017 Evaluation of lipid biosynthesis ability by *Rhodotorula* and *Sporobolomyces* strains in medium with glycerol. Eur Food Res Technol 243:275–286. doi:10.1007/s00217-016-2742-9.

[4] Ju LY, Choi YR, Lee SY, Park JT, Shim JH, Park KH, Kim JW 2011 Screening wild yeast strains for alcohol fermentation from various fruits. Mycobiology 39:33–39. 22783070 10.4489/MYCO.2011.39.1.033 PMC3385091

[5] de Llanos Frutos R, Fernández-Espinar MT, Querol A 2004 Identification of species of the genus *Candida* by analysis of the 5.8S rRNA gene and the two ribosomal internal transcribed spacers. Antonie Van Leeuwenhoek 85:175–185. doi:10.1023/B:ANTO.0000020154.56649.0f. 15028869

[6] Li R, Zhu H, Ruan J, Qian W, Fang X, Shi Z, Li Y, Li S, Shan G, Kristiansen K, Li S, Yang H, Wang J, Wang J 2010 *De novo* assembly of human genomes with massively parallel short read sequencing. Genome Res 20:265–272. doi:10.1101/gr.097261.109. 20019144 PMC2813482

[7] Li R, Li Y, Kristiansen K, Wang J 2008 SOAP: short oligonucleotide alignment program. Bioinformatics 24:713–714. doi:10.1093/bioinformatics/btn025. 18227114

[8] Kimura M 1980 A simple method for estimating evolutionary rates of base substitutions through comparative studies of nucleotide sequences. J Mol Evol 16:111–120. doi:10.1007/bf01731581. 7463489

[9] Kumar S, Stecher G, Li M, Knyaz C, Tamura K 2018 MEGA X: Molecular Evolutionary Genetics Analysis across computing platforms. Mol Biol Evol 35:1547–1549. doi:10.1093/molbev/msy096. 29722887 PMC5967553

